# Fe_2_O_3_ and Gd_2_O_3_ nanoparticles loaded in mesoporous silica: insights into influence of NPs concentration and silica dimensionality

**DOI:** 10.1039/c8ra05576a

**Published:** 2019-01-28

**Authors:** V. Zeleňák, A. Zeleňáková, O. Kapusta, P. Hrubovčák, V. Girman, J. Bednarčík

**Affiliations:** Department of Inorganic Chemistry, Faculty of Sciences, P. J. Safarik University in Kosice Moyzesova 11 04054 Košice Slovakia; Department of Condensed Matter Physics, Faculty of Sciences, P. J. Safarik University in Kosice Park Angelinum 9 04054 Košice Slovakia adriana.zelenakova@upjs.sk; DESY-Hasylab Notkestrasse 85 Hamburg Germany; Frank Laboratory of Neutron Physics, JINR Dubna Russia

## Abstract

Fine Fe_2_O_3_ and Gd_2_O_3_ magnetic nanoparticles (NPs) with sizes 7 nm and 10 nm embedded into mesoporous silica have been prepared using a wet-impregnation method. A comparative study of the reactant concentration along with the hosting matrix symmetry on mesostructuring and the magnetic properties of the nanocomposites have been investigated. Reactants with four different concentrations of Fe^3+^ and Gd^3+^ ions and silica matrices with two different kinds of symmetry (hexagonal and cubic) have been utilized for the study. The structural characterization of the samples has been carried out by the N_2_ adsorption/desorption method, high-energy X-ray diffraction (HE-XRD), TG/DTA, and high resolution transmission electron microscopy (HRTEM). The magnetic properties of the nanocomposites have been examined by means of SQUID magnetometry. It has been found that a range of different magnetic states (diamagnetic, paramagnetic, ferromagnetic, superparamagnetic) can be induced by the feasible tailoring of the particle concentration, the porous matrix symmetry and the composition. Furthermore, the existence of a “critical concentration limit” for embedding the particles within the body of the matrix has been confirmed. Exceeding the limit results in the expulsion of nanoparticles on the outer surface of the mesoporous matrix. Revelation of the relationships between particle concentration, matrix symmetry and magnetic properties of the particular composite reported in this study may facilitate the design and construction of advanced intelligent nanodevices.

## Abstract 2

The concentration of nanoparticles inside the pores and the symmetry of the porous matrix significantly affected the magnetic properties.

## Graphical abstract

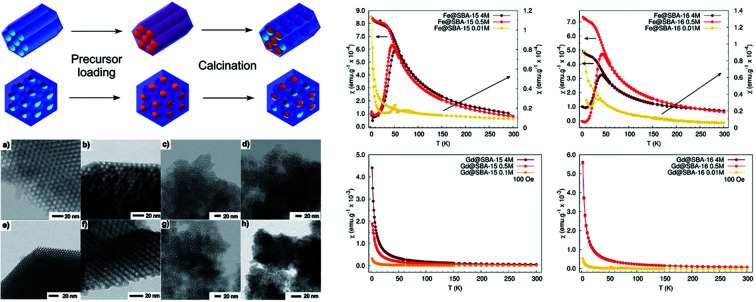

## Introduction

Novel magnetic composite materials based on mesoporous silica have found applications in the fields of catalysis, adsorption, chromatography, chemical sensors and biomedicine.^1–6^ Particularly in biomedicine, nanocomposites consisting of mesoporous silica loaded with magnetic NPs and specific drugs appear very promising for diagnostic and therapeutic applications.^7–10^ The employment of magnetic resonance imaging, hyperthermia treatment or hi-tec stimuli-responsive targeted drug delivery has improved dramatically with the introduction of these kinds of systems. Undoubtedly, one of the most valuable benefits of these materials is their versatility. It stems from their specific inner structure and design. Crucial hosting matrix characteristics like its symmetry and pore size, volume and area are easily tunable during the fabrication process. This enables the further introduction of other structures (*e.g.* nanoparticles, drug molecules) of the desired size into the matrix body. On the other hand, durability, high thermal stability and low toxicity are the qualities that amorphous silica preserves over a broad range of conditions.

Several preparation methods including the one-pot synthesis, wet-impregnation or *in situ* methods have been reported for mesoporous silica based magnetic composite materials. Zhao *et al.*^11^ have developed an *in situ* synthesis of magnetic mesoporous silica *via* a sol–gel process with subsequent precipitation and oxidation. An iron precursor (NH_4_)Fe(SO_4_)_2_·6H_2_O has been utilized in order to obtain Fe_3_O_4_ nanocomposites. The final materials exhibited a pore volume density of 0.64–0.96 cm^3^ g^−1^, a high saturation magnetization value 1.11–5.77 emu g^−1^ and a high adsorption capacity (up to 212 mg g^−1^ for lysozyme) depending on the amounts of the reactants. A magnetic mesoporous silica composite has been fabricated by a sol–gel method in a nitrogen atmosphere where iron containing molecules are dissolved to form the sol into which the mesoporous matrix is subsequently merged.^11^ The structural changes in the regular host matrix body in response to the incorporation of iron oxide nanoparticles (into the vacant mesopores) is evidenced by the decrease in the diffraction intensity observed in the XRD spectra. Another community of authors reported on the wet impregnation method^12,13^ for the preparation of mesoporous silica containing magnetic nanoparticles. Here, metal nitrate solution is mixed with a porous matrix and allowed to dry. Afterwards, the product is calcinated in air. In the case of iron nitrate utilization and calcination in an oxygen atmosphere, Fe_2_O_3_ NPs are formed. Jin *et al.*^14^ synthetized a magnetic composite *via* the pH-adjusting method. They added iron salt solution into the matrix reaction mixture and the adjusted pH value to 7 changing Fe(NO_3_)_3_ into Fe(OH)_3_. The above described methods advantage are control of the magnetic nanoparticle size, use as a nanoreactor, however, the nanoparticles fill the pores and reduce the adsorption capacity of other molecules limiting the size of molecules which can be loaded into the pores to the pores diameter. An important advantage of all the methods mentioned above is the ability to control the size of the nanoparticles. The regular pores of the matrix serve as nanoreactors which constrain the dimensions of the structures embedded inside. Since the pore volume is strictly limited, its occupation by the nanoparticle significantly reduces its adsorption capacity, and the shape and size of other molecules.^15^ Silica nanoparticles doped with gadolinium oxide exhibit promising application potential in biomedicine. Apart from low cytotoxicity of the gadolinium content responsible for the enhanced paramagnetic effect in proton paramagnetic resonance, another quality can be attributed to the system; after specific modification (3-aminopropyltrimethoxysilane), DNA molecules are allowed to bind to the particles' surface electrostatically. The combination of these properties favors the utilization of the system as an imaging agent or for targeted drug delivery.^3^

Similar materials, however, containing NPs on the basis of iron, are adept for hyperthermia treatment.^17^ Wang *et al.*^16^ reported the fabrication of silica NPs with large pores, where the co-precipitation method was employed for Fe_3_O_4_ NPs introduction.

A host of studies devoted to particular magnetic composites containing nanoparticles of iron and gadolinium oxides has been reported. However, work dealing with their systematic comparison based on an analysis of the hosting matrix symmetry along with the particles' composition and concentration has not yet been performed.

Hence, our objective was to design and examine a variety of systems from the structural and magnetic point of view. The wealth of experimental data was further analyzed with the aim to find general rather than specific features and trends within the similar systems of the series. For the purpose of the study, a total set of 16 samples were prepared. Namely, nanoparticles of Gd_2_O_3_ and Fe_2_O_3_ were introduced in four concentrations into the silica matrices of the two different symmetries.

## Experimental

### Materials

All chemicals: 35% HCl, 98% TEOS – tetraetoxysilane, Pluronic P-123, Pluronic F-127, 99.9% Fe(NO_3_)_3_·9H_2_O, 99.9% Gd(NO_3_)_3_·6H_2_O were purchased from Sigma-Aldrich and were used without further purifications. Deionized water was used in all experiments.

### Preparation of blank mesoporous matrices

The SBA-15 mesoporous matrix with a hexagonal symmetry (*P*6*mm*) was prepared following the procedure described by Zhao *et al.*^18^ The synthesis was performed in a molar ratio: 1TEOS : 5.9HCl : 193H_2_O : 0.017 P-123. 30 g distilled water was mixed with 120 g 2 M HCl in polypropylene beaker and stirred at 400 rpm at 35 °C. 4 g P-123 was added into the reaction mixture. After Pluronic dissolution, 8 g TEOS was added into the beaker. The reaction mixture was stirred continuously for 24 hours (400 rpm, 35 °C). Further, the mixture was aged in an oven for 24 hours at 80 °C. Later the mixture was washed with distilled water. The acquired white powder was kept aging, the product was filtered under vacuum and dried in the air at room temperature. Finally, the dried powder was calcinated in an air atmosphere at 500 °C for 7 hours.

The mesoporous matrix SBA-16 with a cubic symmetry (*Im*3̄*m*) was prepared by the procedure described by Kim *et al.*^19^ The molar ratio of reactants was: 1TEOS : 0.4HCl : 144H_2_O : 0.0016 P-123 : 0.0037 F-127. 20 g of distilled water was mixed with 92.7 g HCl in a polypropylene beaker, subsequently, 0.38 g P-123 and 1.9 g F-127 were added and stirred at 400 rpm and 35 °C. After the dissolution of both Pluronics, 8.5 g TEOS was added dropwise. This mixture was stirred at constant conditions (400 rpm, 35 °C) for 15 minutes. Further, the mixture was kept ageing in an oven at 100 °C for 24 hours. The final product was filtered, several times and washed with ethanol and allowed to dry at ambient temperature. After drying, white powder was calcinated at 500 °C in air atmosphere for 7 hours.

### Modification of mesoporous matrices by iron and gadolinium precursors

Prepared mesoporous matrices SBA-15 and SBA-16 were modified employing the wet impregnation method utilizing Fe(NO_3_)_3_ and Gd(NO_3_)_3_ solutions with different concentrations (0.01 M; 0.1 M; 0.5 M and 4 M). 250 mg of the porous matrix (SBA-15/SBA-16) was mixed with the corresponding solution by means of ultrasonication at 50 °C during 30 min. The obtained solutions were centrifuged at 2000 rpm for 10 min and the product was separated and dried in an oven at 80 °C. The dried powders were calcinated at 500 °C for 7 hours. At the end of the process, 16 different nanocomposite samples were prepared and denoted as B@SBA-*X Y*M (B = Fe/Gd, *X* = 15/16, *Y* = 0.01; 0.1; 0.5 and 4).

### Experimental methods

As prepared and calcinated mesoporous matrices were characterized by infrared spectra using Nicolet 6700 FT-IR instrument at ambient temperature with the following settings: transmission mode, a range of 300–4000 cm^−1^ (32 scans), KBr technique.

Blank as well as modified calcinated samples were examined by the nitrogen adsorption/desorption volumetric method at 77 K utilizing Nova 1200e Quantachrome analyzer. Samples were degassed during 8 hours at 420 K before measurement. This method provided information on the samples' surface area (BET method), external surface area (*t*-plot method), pore diameter and pore volume (DFT method).

X-ray powder diffraction was used for phase/composition analysis of nanoparticles embedded inside the porous matrices. Diffraction measurements were carried out by synchrotron radiation of energy 60 kV and wavelength *λ* = 0.0207 nm at PETRA III accelerator at DESY, Hamburg. Capton capillaries were filled with powder samples and the scattering intensity was measured as a function of the scattering vector, *q*, being defined as *q* = (4π/*λ*)sin *θ*, where 2*θ* is the scattering angle. The obtained diffraction patterns were processed *via* FIT2D software employing CeO_2_ as a calibration standard. Particles' size was determined by Scherer formula.

Prepared systems were also examined by TEM (Transmission electron microscopy) with a JEOL 2100 operating at 200 kV in STEM mode. Powder samples were dispersed by means of ultrasonication in methanol and added to carbon coated copper grids of mesh size 200.

Magnetic measurements were performed by MPMS 5XL SQUID based magnetometer from Quantum Design. Static dc-magnetizations were recorded in the temperature range 2–300 K in zero-field-cooling (ZFC) and field-cooling (FC) protocols. Magnetization *vs.* applied field (magnitude) loops were measured at 2 K and 300 K up to 50 000 Oe. In order to determine coercive field, each sample was thermally demagnetized by heating to room temperature followed by cooling the sample in the absence of an applied field down to the measuring temperature 2 K or 300 K.

## Results and discussion

**Scheme 1:**
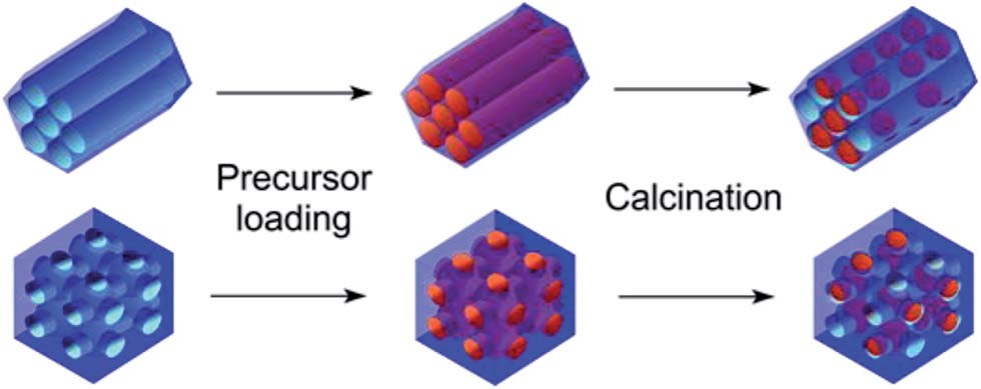
Schematic of samples' preparation using the wet-impregnation method.

The general idea of particles' introduction into the hollow matrices is illustrated in Scheme 1. The combination of two different metal ions (Gd^3+^ or Fe^3+^), a pair of matrices with 2D-hexagonal or 3D-cubic symmetry and four different nanoparticle concentrations resulted in the preparation of the series of 16 nanocomposite samples.

**Fig. 1:**
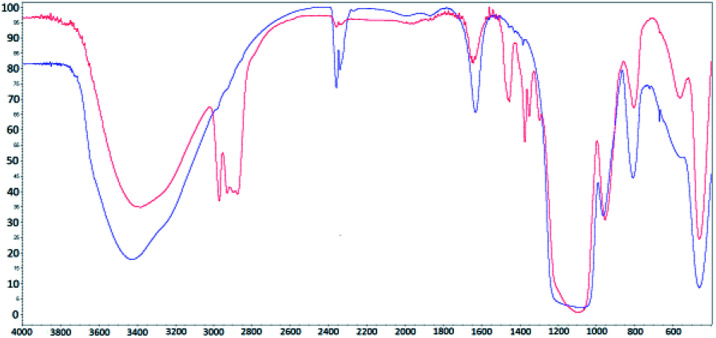
IR spectra for synthesized (red line) and calcinated (blue line) SBA-15 matrix.

FT-IR spectra for the synthesized (red) and calcinated (blue) SBA-15 matrix are shown in Fig. 1. In both IR spectra, (for calcinated as well as synthesized) the O–H bond valence vibration at ∼3400 cm^−1^ from silanol Si–O–H bonds can be recognised. The presence of the organic surfactant is confirmed by C–H bond valence vibrations between 3000–2800 cm^−1^ and deformation vibrations between 1450–1350 cm^−1^. These vibrations are absent in the calcinated sample spectra indicating surfactant removal from the pores. Characteristic signals of Si–O–Si bonds are present at 1094 cm^−1^ and 964 cm^−1^ for valence vibrations and at 800 cm^−1^ and 460 cm^−1^ for the deformation vibration. The occurrence of peaks at 1635 cm^−1^ and between 2360–2340 cm^−1^ is attributed to water and carbon dioxide, respectively. These molecules were absorbed from air and their presence is evidence of empty pores in the calcinated matrix.

**Fig. 2:**
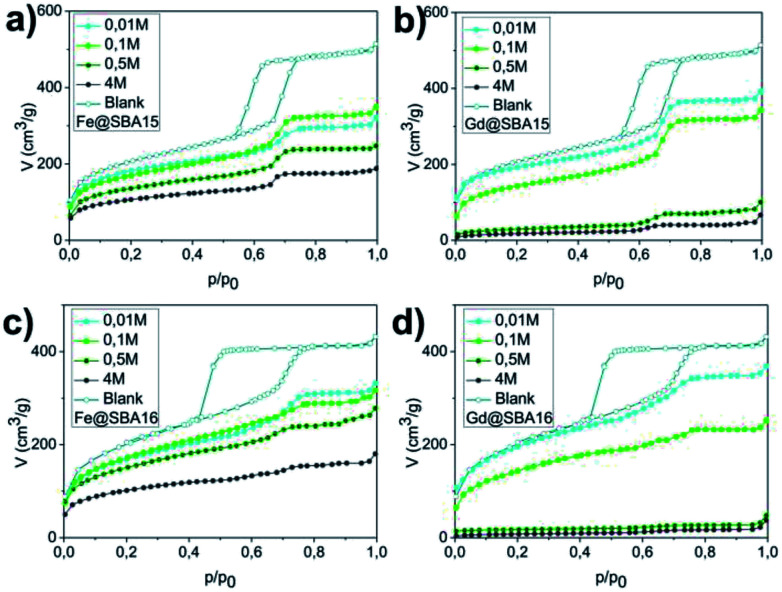
Sorption graphs for all series of samples: (a) hexagonal composites containing Fe^3+^ (Fe@SBA-15 *Y*M), (b) hexagonal composites containing Gd^3+^ (Gd@SBA-15 *Y*M), (c) cubic composites containing Fe^3+^ (Fe@SBA-16 *Y*M), (d) cubic composites containing Gd^3+^ (Gd@SBA-16 *Y*M).

The pore volume, diameter and surface area were established by the nitrogen adsorption/desorption method at 77 K. Fig. 2 shows adsorption isotherms for each sample. For clarity, only absorption isotherms are displayed for the modified samples. Hysteresis loops of IVa type, characteristic of a mesoporous material SBA-15, are shown in Fig. 2(a) and (b), while curves in Fig. 2(c) and (d) correspond to the hysteresis loops of IVb type and are typical of the mesoporous material SBA-16. The absorbed nitrogen volume decreases with as the precursor concentration increase for both SBA-15 and SBA-16 matrices. One can also note significant difference between the samples modified by iron and those modified by gadolinium precursor.

**Fig. 3:**
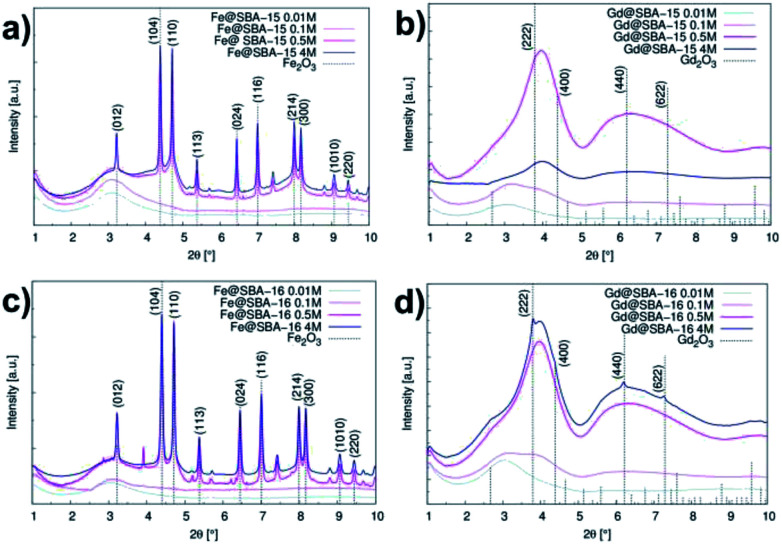
Intensity *vs.* scattering angle in samples series: (a) hexagonal composites containing Fe^3+^ (Fe@SBA-15 *Y*M), (b) hexagonal composites containing Gd^3+^ (Gd@SBA-15 *Y*M), (c) cubic composites containing Fe^3+^ (Fe@SBA-16 *Y*M), (d) cubic composites containing Gd^3+^ (Gd@SBA-16 *Y*M).

For the cases of higher precursor concentrations (0.5 M and 4 M), the adsorbed nitrogen volume significantly decreases when comparing samples containing Gd^3+^ to the samples containing Fe^3+^. This indicates that after calcination, the pores show a higher propensity for filling by gadolinium oxide than by iron oxide. The data from the adsorption measurements were utilized for calculation of the specific adsorption parameters (specific surface area *S*_BET_, pore diameter *d*_DFT_ and pore volume *V*_DFT_) for all 16 studied samples. The complete summary of the values established for the nanocomposites containing Fe^3+^ and Gd^3+^ are shown in Tables 1 and 2, respectively. With the aim of determining the composition and structural phases of nanoparticles embedded inside the matrices, high energy X-ray diffraction (HE-XRD) experiments using synchrotron radiation were performed. The diffraction patterns of the nanocomposites are demonstrated in Fig. 3. For all samples modified with iron precursor, (Fig. 3(a) and (c)) exhibit one broad peak at 2*θ* = 3.09° assigned to the mesoporous silica matrix. Samples with a higher concentration of Fe^3+^ ions (*Y*M = 0.5 M a 4 M) show reflections for α-Fe_2_O_3_ (hematite, space group *R*3̄*c* (no. 167), JCPDS no. 86–0550).

**Table 1:** Textural parameters of mesoporous composites containing Fe_2_O_3_ NPs

Sample	*S*_BET_ (cm^2^ g^−1^)	*d*_DFT_ (Å)	*V*_DFT_ (cm^3^ g^−1^)
SBA-15 (blank)	708.18	97.73	0.73
SBA-16 (blank)	699.42	104.8	0.64
Fe@SBA-15 0.01 M	629.21	94.16	0.52
Fe@SBA-15 0.1 M	538.50	94.16	0.48
Fe@SBA-15 0.5 M	429.45	94.16	0.36
Fe@SBA-15 4 M	331.36	94.16	0.26
Fe@SBA-16 0.01 M	282.68	25.83	0.46
Fe@SBA-16 0.1 M	263.40	25.83	0.43
Fe@SBA-16 0.5 M	468.86	25.83	0.38
Fe@SBA-16 4 M	322.86	25.04	0.24

**Table 2:** Textural parameters of mesoporous composites containing Gd_2_O_3_ NPs

Sample	*S*_BET_ (cm^2^ g^−1^)	*d*_DFT_ (Å)	*V*_DFT_ (cm^3^ g^−1^)
Gd@SBA-15 0.01 M	503.56	84.62	0.57
Gd@SBA-15 0.1 M	493.45	84.62	0.48
Gd@SBA-15 0.5 M	100.53	84.62	0.11
Gd@SBA-15 4 M	61.59	84.62	0.06
Gd@SBA-16 0.01 M	619.40	25.83	0.52
Gd@SBA-16 0.1 M	513.39	25.83	0.35
Gd@SBA-16 0.5 M	96.27	25.83	0.09
Gd@SBA-16 4 M	27.37	25.83	0.03

**Fig. 4:**
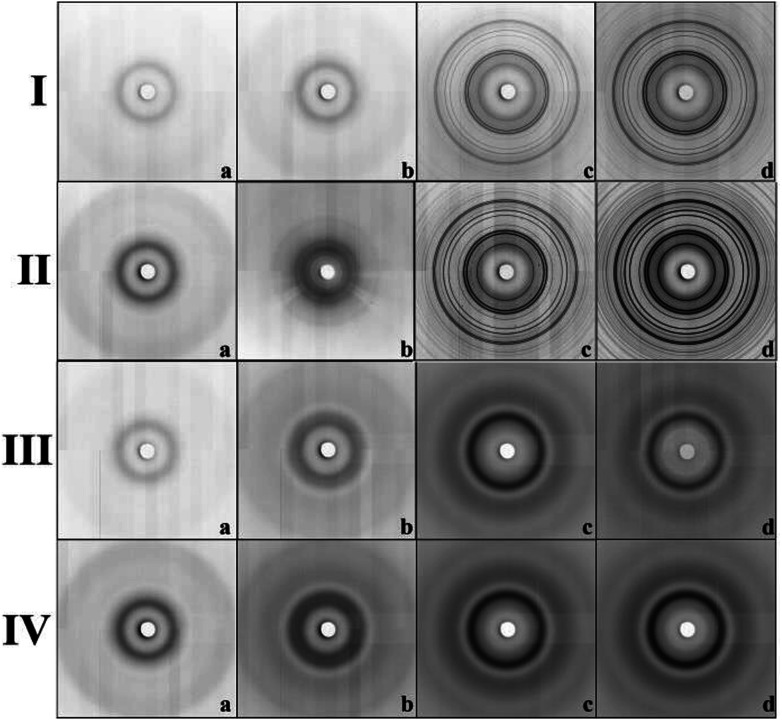
Diffraction patterns of samples' series: (I) Fe@SBA-15, (II) Fe@SBA-16, (III) Gd@SBA-15, (IV) Gd@SBA-16. Concentration of NPs: (a) 0.01 M; (b) 0.1 M; (c) 0.5 M and (d) 4 M, respectively.

The expected change of the patterns was observed by gradually increasing the iron(iii) precursor concentration (*Y*M = 0.01 M; 0.1 M; 0.5 M and 4 M) (Fig. 4I and II). At lower concentrations (*Y*M = 0.01 M; 0.1 M), the patterns point to the amorphous character of the samples. This confirms that the hematite nanoparticles exclusively occupy the internal surface of the pores. The diffraction signal from nanoparticles is shielded by the surrounding amorphous silica matrix and due to this, only diffusive patterns with no evidence of crystalline phases were detected in the samples of Fe@SBA-*X Y*M (*X* = 15/16, *Y* = 0.01; 0.1).

At higher concentrations of Fe^3+^ ions (0.5 M and 4 M), the presence of a crystalline phase was detected as shown in Fig. 4(Ic, Id, IIc and IId). We assume that due to the relatively high precursor concentration in the silica matrix pores, the volume capacity of the pores was not sufficient for confinement of the progressive growth of the Fe_2_O_3_ NPs. As a consequence, the excess precursor runs over the pores and the particles also formed one external silica surface. This could rationalize the detection of the hematite phase with high crystallinity. Diffraction patterns of the samples with a higher concentration of iron precursor which (*Y* = 0.5 M; 4 M) show clear evidence of the crystalline phase, and were further used for the estimation of the average particle size *via* Scherrer formula.^20^

The hematite nanoparticles had average sizes of *D* = 16.8 nm and *D* = 18 nm for the Fe@SBA-15 and Fe@SBA-16 samples with elevated precursor concentration, respectively, and were found to be significantly higher than the pore sizes of both silica matrices. This supports the assumption of particle formation out the pores as discussed above.

Intensity *vs.* scattering angle of the samples containing Gd^3+^ nanoparticles is demonstrated on Fig. 3(b) and (d). All the samples exhibit a broad peak in the vicinity of 2*θ* = 3.09°, while the samples with a higher concentration of gadolinium nanoparticles (0.5 M a 4 M) are characteristic of the additional broad peak occurring at 2*θ* = 6.2°. All of these features are assigned to mesoporous matrix with the amorphous nature. In spite of the progressive abundance of nanoparticles in the matrices, only weak peaks corresponding to Gd_2_O_3_ (space group *Ia*3̄ (no. 206), JCPDS no. 43-1014) phase are observed in the diffraction patterns. We assume that almost all diffraction peaks assigned to Gd_2_O_3_ are hidden under the broad peaks of the silica matrix. Diffusive patterns typical of all samples containing Gd^3+^ NPs, Fig. 4III(a–d) and IV(a–d) point to differences between the nanocomposites modified by iron and gadolinium, even though they are prepared with the same concentration and silica matrix symmetry. This suggests that the mechanism of impregnation and growth of the nanoparticles in the mesopores is rather different in the case of Fe^3+^ and Gd^3+^ ions.

**Fig. 5:**
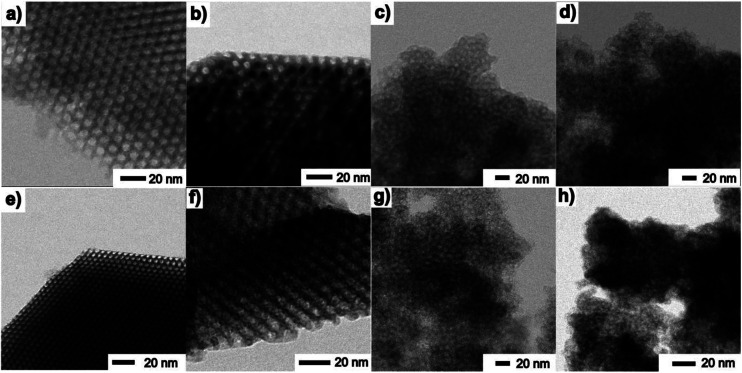
TEM images of samples: Fe@SBA-15 (a) lowest and (b) highest concentration of NPs, Fe@SBA-16 (c) low and (d) high concentration of NPs, Gd@SBA-15 (e) low and (f) high concentration of NPs, Gd@SBA-16 (g) low and (h) high concentration of NPs.

**Fig. 6:**
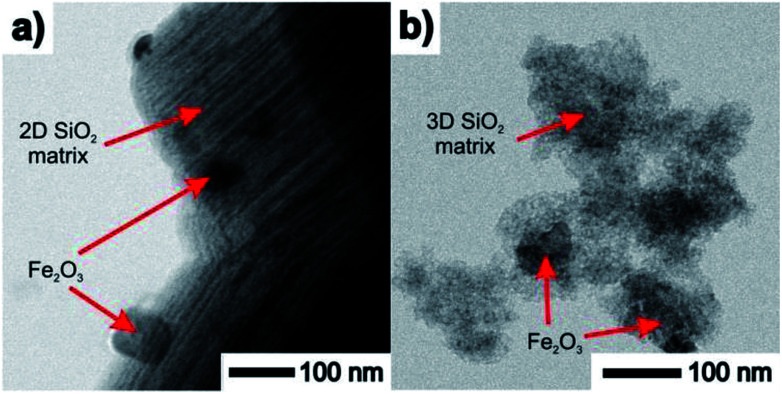
TEM images of samples containing the highest concentration of Fe^3+^ ions with large particles at the surface. (a) 2D nanocomposite Fe@SBA-15 (hexagonal symmetry), (b) 3D nanocomposite Fe@SBA-16 (cubic symmetry).

TEM images taken in the STEM mode are shown in Fig. 5 and 6. The process of progressive pore filling with increasing concentration of Fe^3+^ or Gd^3+^ precursors is represented by pronounced darkness of the corresponding area. In the case of Fe@SBA-15 nanocomposite with the highest ion concentration, spherical Fe_2_O_3_ NPs confined by cylindrical pores of hexagonal arrangement are clearly recognized. On the other hand, a similar contrast between the nanoparticles and silica matrix in the Gd_2_O_3_ nanocomposites was not observed. A detailed TEM study of all the nanocomposite samples show that a small portion of the larger particles are present on the matrices' external surface at the highest concentration of Fe_2_O_3_ nanoparticles in the 2D (SBA-15) and 3D (SBA-16) matrices as seen in Fig. 6. This finding is in accordance with the results of the XRD analysis and both support the assumption of the existence of a “critical concentration”. Apparently, with increasing concentration of Fe^3+^ precursor, the critical limit can be exceeded when the particles are not only formed inside of pores, but are also expelled out from the porous system. We suppose that the strong XRD signal documented for Fe@SBA-15 and Fe@SBA-16 with NPs concentrations *Y* = 0.5 M and *Y* = 4 M, see Fig. 3(a), (c) and 4(Ic, Id, IIc and IId) comes from the crystalline phase located on the external surface of the matrices. A similar process of NPs expulsion on the external surface of the nanocomposites containing Gd_2_O_3_ NPs was not observed.

**Fig. 7:**
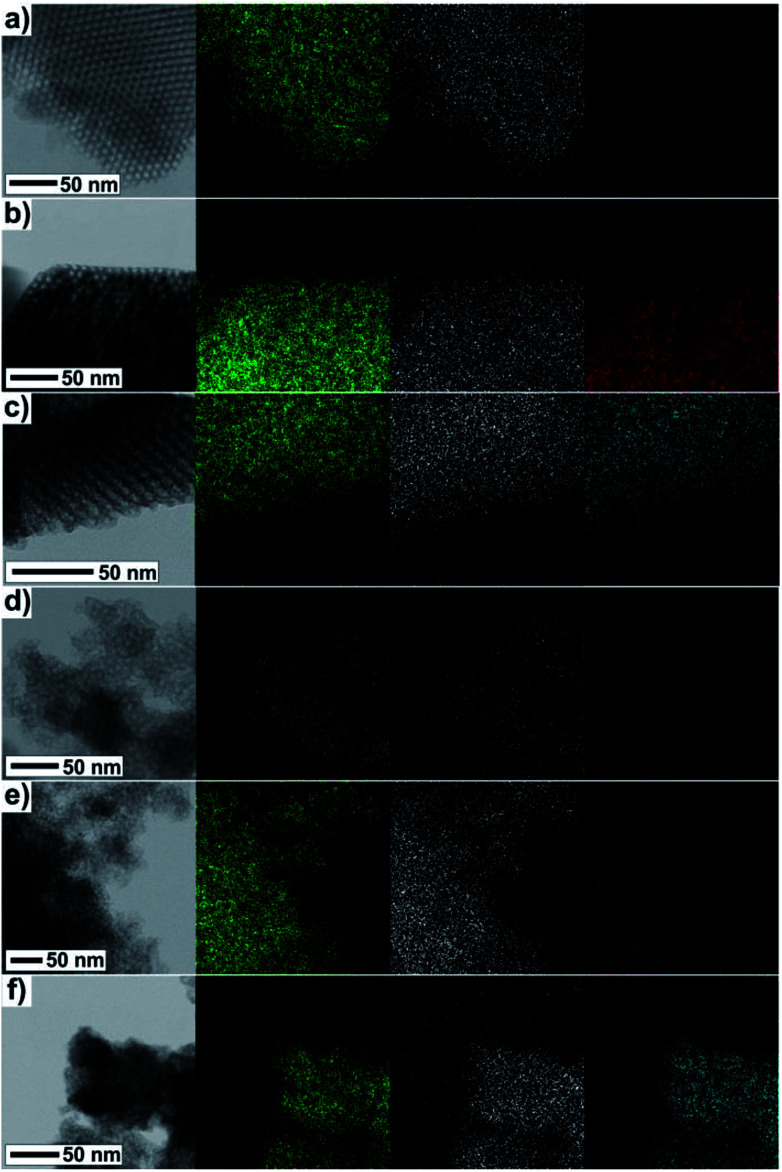
TEM and corresponding EDS images of samples (a) Fe@SBA-15 *Y* = 0.01 M; (b) Fe@SBA-15 *Y* = 4 M; (c) Gd@SBA-15 *Y* = 4 M, (d) Fe@SBA-16 *Y* = 0.01 M; (e) Fe@SBA-16 *Y* = 4 M, (f) Gd@SBA-16 *Y* = 4 M. Different colours represent different atoms: Si – green, O – white, Fe – red, Gd – cyan.

The direct evidence of progressive filling of the silica pores depending on the increasing concentration of Fe^3+^ and Gd^3+^ precursors is demonstrated by the TEM-EDS measurements, see Fig. 7. The very scarce but explicit presence of Fe^3+^ and Gd^3+^ ions was documented unambiguously even in samples with the lowest concentration of metal precursors, Fig. 7(a) and (d). Further, the density of points along with the (colour) contrast were found to be gradually enhanced for the sample series with the increasing concentration of the metal precursors, revealing the progressive incorporation of the NPs into the matrix pores.

**Fig. 8:**
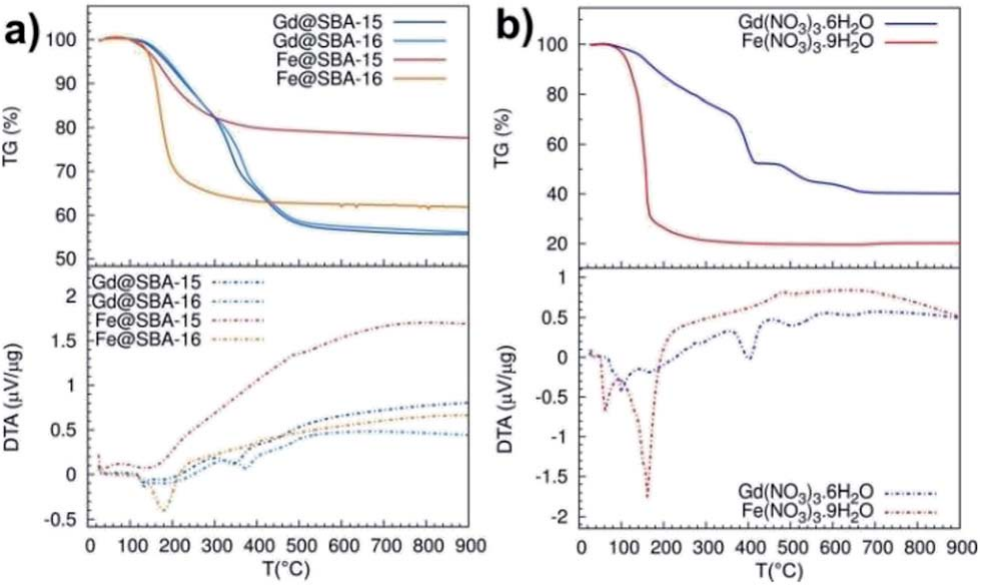
TG (solid line)/DTA (dashed line) curves measured in argon atmosphere: (a) pure metal precursors, (b) porous samples with NPs; Gd@SBA-15 (dark blue), Gd@SBA-16 (light blue), Fe@SBA-15 (red), Fe@SBA-16 (orange).

A deeper investigation of the differences between Fe^3+^ and Gd^3+^ samples along with the elucidation of the particle's growth mechanism was carried out by means of thermal analysis experiments, see Fig. 8. At first, we studied thermal decomposition of four mesoporous samples SBA-15 and SBA-16 impregnated by Fe(NO_3_)_3_·9H_2_O and Gd(NO_3_)_3_·6H_2_O, Fig. 8(a).

While the DTA/TG curves of Gd@SBA-15 (dark blue line) and Gd@SBA-16 (light blue line) are almost identical and both point to sample decomposition in three subsequent steps, the decomposition of the samples Fe@SBA-15 (red line) and Fe@SBA-16 (orange line) proceed with only one sharp observed peak. Moreover, differences in the decomposition process of samples containing Fe_2_O_3_ NPs loaded in the SiO_2_ matrix with different symmetries of SBA-15 (Fe@SBA-15) and SBA-16 (Fe@SBA-16) were confirmed. This is due to differences in the symmetry and pore sizes of SBA-15 (hexagonal) and SBA-16 (cubic symmetry).

Thermal decomposition of the pure nitrate salts, Fe(NO_3_)_3_·9H_2_O and Gd(NO_3_)_3_·6H_2_O, which serve as metal precursors was also examined, see Fig. 8(b). While Fe(NO_3_)_3_·9H_2_O (red line) decomposes in one sharp step in the temperature range 130–180 °C, the decomposition of Gd(NO_3_)_3_·6H_2_O (blue line) is slower and it takes place in three steps in the temperature range 100–530 °C.

Taking into account the results of the DTA/TG analysis, the XRD and TEM observations can be explained. When the concentration of the Gd or Fe salts is low during decomposition of the nitrates in the pores of the silica matrix, the released molecules of water or nitrogen oxide can freely diffuse from the pores and during decomposition, and the formation of the corresponding oxides Fe_2_O_3_ or Gd_2_O_3_ takes place. When the concentration of the salts increases, the nanoparticles with the higher concentration start to fill the pores and the release of gases during the decomposition of the salts from the pore system is more difficult.

On the contrary, in the case of Gd(NO_3_)_3_·6H_2_O the decomposition is slow and the gases are allowed to diffuse slowly out of the pores. However, in case of the Fe(NO_3_)_3_·9H_2_O which decomposes in one fast step, the partial pressure inside of the pores is so high that the nanoparticle plugs which block the pores are pushed out onto the external surface of the silica.

Assuming this scenario, the observation of nanoparticles on the silica surface documented in the TEM pictures of Fe@SBA-15 4 M, see Fig. 6, and their absence in the case of Gd@SBA-15 4 M can be clarified.

**Fig. 9:**
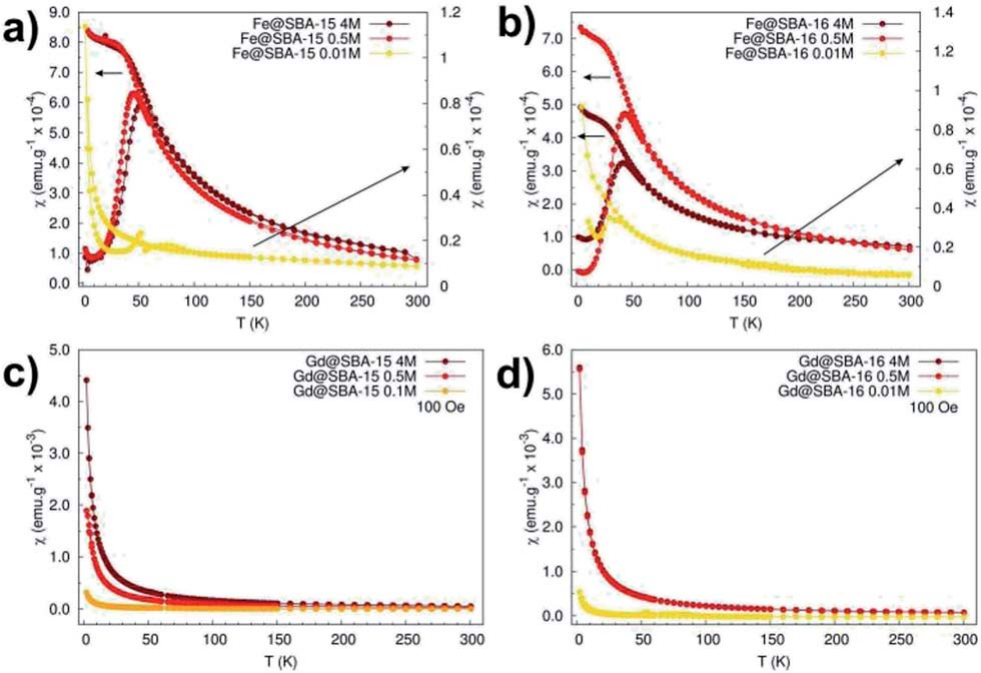
Magnetic dc-susceptibility *vs.* temperature dependences measured in ZFC/FC protocols in external magnetic field of 100 Oe. (a) Hexagonal composites with different concentration of Fe^3+^ NPs, (b) cubic composites with different concentration of Fe^3+^ NPs, (c) hexagonal composites with different concentration of Gd^3+^ NPs, (d) cubic composites with different concentration of Gd^3+^ NPs.

The magnetic properties of all the samples were also scrutinized in order to recognise and highlight differences between them. Susceptibility and magnetization dependences on the temperature and applied field were recorded and compared for this purpose. Fig. 9 shows the temperature dependence of the magnetic dc-susceptibility of the samples obtained in the ZFC and FC protocols.

The nanocomposites containing iron oxide (Fig. 9a and b) exhibit the hallmarks of superparamagnetic systems: (i) the presence of a maximum in the ZFC curves at the blocking temperature *T*_B_ (*T*_B_ ∼45 K), (ii) the merging of the ZFC/FC curves above the blocking temperature. On the other hand, the samples containing Gd^3+^ NPs manifest paramagnetic behaviour that is typical of almost all Gd^3+^ salts,^21^ bulk and Gd_2_O_3_ particles^22^ at room temperature. These samples also show enhanced susceptibility with increased nanoparticles' concentration (Fig. 9c and d). Intriguingly, this dependence was not broken in the case of samples containing Fe^3+^ NPs. As it is seen in Fig. 9a and b, nanocomposites with Fe_2_O_3_ NPs loaded in both 2D (Fe@SBA-15, see Fig. 9a) and 3D (Fe@SBA-16, see Fig. 9b) matrices are characteristic of lower susceptibility values when compared the highest (4 M, dark red) to the lowest (0.5 M, red) concentration. This most likely points to the existence of a “critical” Fe_2_O_3_ NPs concentration at which the internal porous system is optimally filled by the particles. Above this “critical” concentration, a portion of NPs is ejected out of the internal surface. Since the growth mechanism of NPs is controlled by the size of the internal porous system, the Fe_2_O_3_ particles located out of pores are allowed to exhibit larger sizes (about 80 nm, see Fig. 6). However, the critical size for superparamagnetic particles of Fe_2_O_3_ is 35 nm,^23^ NPs exceeding this limit are not in a superparamagnetic state. As a consequence, the susceptibility is reduced in samples with the highest concentration of Fe_2_O_3_ NPs (Fe@SBA-15 4 M and Fe@SBA-16 4 M), Fig. 9a and b.

**Fig. 10:**
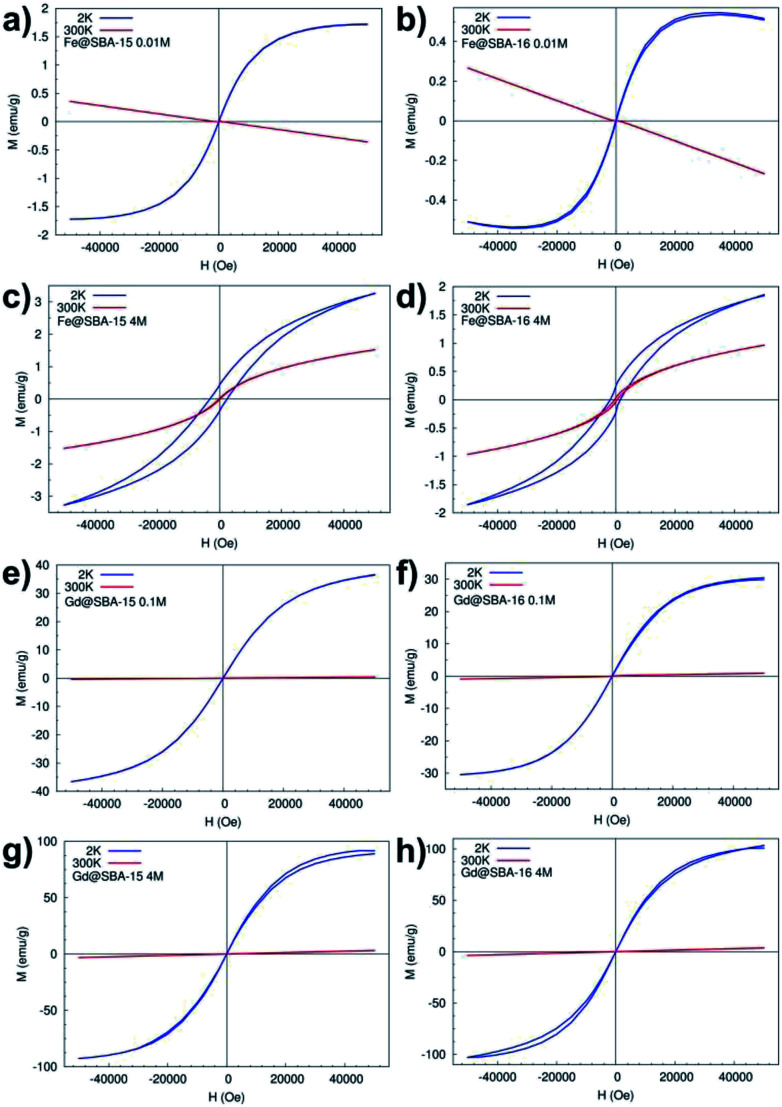
*M*(*H*) curves measured at 2 K and 300 K: (a) hexagonal and (b) cubic nanocomposites with low concentration of Fe^3+^ NPs, (c), hexagonal and (d) cubic nanocomposites with high concentration of Fe^3+^ NPs, (e) hexagonal and (f) cubic nanocomposites with low concentration of Gd^3+^ NPs, (g) hexagonal and (h) cubic nanocomposites with high concentration of Gd^3+^ NPs.

Magnetization curves (*M*(*H*)) measured at temperatures 2 K and 300 K, Fig. 10, confirm the results from temperature dependence of the susceptibility. In all four samples with the lowest concentration of Fe^3+^ and Gd^3+^ NPs *Y* = 0.01 M (namely: Fe@SBA-15 0.01 M, Fe@SBA-16 0.01 M, Gd@SBA-15 0.01 M, Gd@SBA-16 0.01 M) the diamagnetic state was observed at room temperature (300 K) and paramagnetic behaviour appears with decreasing temperature to 2 K due to the thermally activated processes.

In the nanocomposites containing Fe^3+^ NPs with higher concentrations, the samples Fe@SBA-15, Fe@SBA-16, 0.5 M and 4 M (see Fig. 10c and d), typical superparamagnetic behaviour was confirmed as well as from the M(H) loops. At a temperature of 300 K (*T* > *T*_B_ ∼ 45 K), Fig. 10c-red line, 10d-red line, the magnetic moments of Fe_2_O_3_ NPs can freely fluctuate in the external magnetic field leading to a lack of superparamagnetism and coercivity. Below *T*_B_ (*T* < *T*_B_ ∼ 45 K), the magnetic moments are blocked in the external magnetic field direction and coercivity caused by ferromagnetic interaction appears, Fig. 10c-blue line and 10d-blue line.

On the other hand, the samples containing Gd^3+^ NPs Gd@SBA-15 and Gd@SBA-16, Fig. 10g and h, with the same nanoparticle concentration as the samples containing Fe^3+^ NPs show like-paramagnetic behaviour in the temperature range 10–300 K, which was confirmed by the measured M(H) curves (Fig. 10) and the ZFC/FC curves (Fig. 9). The field dependence of the magnetization measured at 2 K in samples with higher concentration of Gd^3+^ NPs also displays a weak “wasp waist”^24^ remanence – free hysteresis, Fig. 10g – blue line, 10h – blue line. This non-typical behaviour indicates that the anisotropy field in the studied Gd^3+^ NPs is larger than 50 000 Oe and may be the result not only on the reduction of the dimensions from the bulk to the nanoscale and a dramatic increase in surface area but also by the associated crystalline lattice expansion. This expansion may result in longer Gd–Gd bonds and weaker ferromagnetic coupling.

## Conclusion

Nanocomposite materials containing Fe_2_O_3_ and Gd_2_O_3_ nanoparticles with the same concentrations were prepared by the nanocasting method. Structural analysis confirms that the nanocasting provides a simple procedure for which the silica matrix serves as a nanoreactor for the growth of the nanoparticles. Temperature and field dependencies of the magnetization of all samples were compared. The composite containing Fe_2_O_3_ nanoparticles show superparamagnetic behaviour with a blocking temperature around 45 K. Otherwise, paramagnetic properties were observed for the sample with Gd_2_O_3_ (above 10 K). Additionally, due to free pores, the silica matrix could serve as a medium for the encapsulation of drugs to create magnetically vectored drug delivery systems.

## Conflicts of interest

There are no conflicts to declare.

## Supplementary Material
